# Pin-Retained Amalgam: An Intricate Restorative Case Study

**DOI:** 10.7759/cureus.64118

**Published:** 2024-07-08

**Authors:** Harshita Dhanrajani, Kajol Relan, Manoj Chandak, Priyanka Paul, Gaurav Hemnani

**Affiliations:** 1 Dentistry, Sharad Pawar Dental College and Hospital, Datta Meghe Institute of Higher Education and Research, Wardha, IND; 2 Conservative Dentistry and Endodontics, Sharad Pawar Dental College and Hospital, Datta Meghe Institute of Higher Education and Research, Wardha, IND; 3 Public Health Dentistry, Sharad Pawar Dental College and Hospital, Datta Meghe Institute of Higher Education and Research, Wardha, IND

**Keywords:** pin-retained amalgam, indirect retention, pins, restoration, amalgam

## Abstract

Amalgam has made dental restorations more manageable, especially in difficult cases, due to its strength, durability, and relatively low cost. There can be pros and cons of each dental material depending on which class of cases it is applied or not applied for, depending on the patient's need, and, of course, the dentist's choice. These materials are used frequently for their hardness and durability and are ideal for extensive restoration procedures. On the other hand, composite resins are considered to be more aesthetic for the profitable areas where esthetics are more important. In contrast, glass ionomer cement is considered to be ideal for low-stress-bearing areas. The following case report of pin-retained amalgam restoration presents an example of its application in practical situations and the factors that must be considered to justify the use of this material against others. This paper explains various factors in considering pin-retained amalgam restorations and the procedure for a better and positive outcome.

## Introduction

One of the oldest and most widely used material for restoration in dentistry is dental amalgam. It has been used in restorative dentistry as a cost-effective, long-lasting, yet secure material for a period of over 150 years [1]. Su Kung (659 AD) mentioned the use of dental amalgam in a mixture [2]. In 1528, Johannes Stoker, a municipal physician in Ulm, Germany, suggested amalgam as a filling material all over Europe [3]. After a lot of experiments and a lot of researches Auguste Taveau (1816) used amalgam with small amount of mercury and melted silver coins; in 1826, this mixture was used as a dental restorative and filler [4].

When a considerable amount of enamel and dentin is removed after cavity preparation it affects the retention of the amalgam. The wearing and tear resistance varies from "good" to "excellent," and this is considered when contributing to the longevity of the dental amalgam restoration. This is a desirable quality because mastication exerts a lot of pressure on the restorations. Additionally, amalgam outlasts glass ionomer, composite, and resin ionomer with a higher duration of about 10 years. A brittle material, amalgam fractures along the edges and has the highest wear resistance among the rest of the repair techniques [5]. When it is not possible to generate sufficient resistance and retention forms using slots, grooves, or undercuts alone, pins are used. Pins are not only used for retention but also for core build up and complex restorations. The purpose of pin placement is to secure restorative material to a prepared tooth cavity. For complex cavity preparation direct retention. For indirect retention, three types of metal pins are available: cemented pins, friction-lock pins, and self-threading pins [6]. The count of pins required should be determined by the extent of the affected tooth [7]. The pin retains the restoration and increases the retention of the prepared tooth. This pin-retained amalgam restoration is done in a single visit. When it comes to retention, amalgam restorations with pins significantly exceed restorations without boxes or restorations that just use bonding techniques.

## Case presentation

A 32-year-old female reported to the Department of Conservative Dentistry and Endodontics, Sharad Pawar Dental College and Hospital, with chief complaints of pain and food lodgment in the lower left back region of the jaw for two weeks. The pain was sharp shooting in nature, aggravated by hot and cold stimuli, and was relieved immediately after the removal of the stimulus. It was not radiated to any other region of the jaw. There was no history of night pain, swelling, or pus discharge. Past medical history was irrelevant. She gave a past dental history of root canal treatment in the lower left back region of the jaw three years back. Extraoral examination showed bilateral symmetry of the jaw. The temporomandibular joint was smooth and synchronous with no clicking sound. The lips were competent. On intraoral examination, all the teeth were present. There were deep occlusal caries seen with 37, which was tender on percussion. A pericoronal flap was seen with 38, a metal crown prosthesis was seen with, 36 and a tooth-colored prosthesis was seen with 35. No sinus tract opening was present.

A neural sensibility test, including a cold test and an electric pulp test, was performed on tooth 37. The cold test, using endo-ice applied to the tooth, gave a response in 4-5 seconds. An electric pulp test was also conducted to assess pulp vitality. For comparison, a contralateral tooth was tested first, and the response for both teeth was near to similar. An intra-oral periapical radiograph was indicated with 37, which showed caries involving enamel and dentin with no involvement of the pulp. No periapical changes were seen in the radiograph. After considering all the clinical and radiographic findings and correlating them with the signs and symptoms as well as the chief complaints, a diagnosis of acute reversible pulpitis with 37 was made.

**Figure 1:**
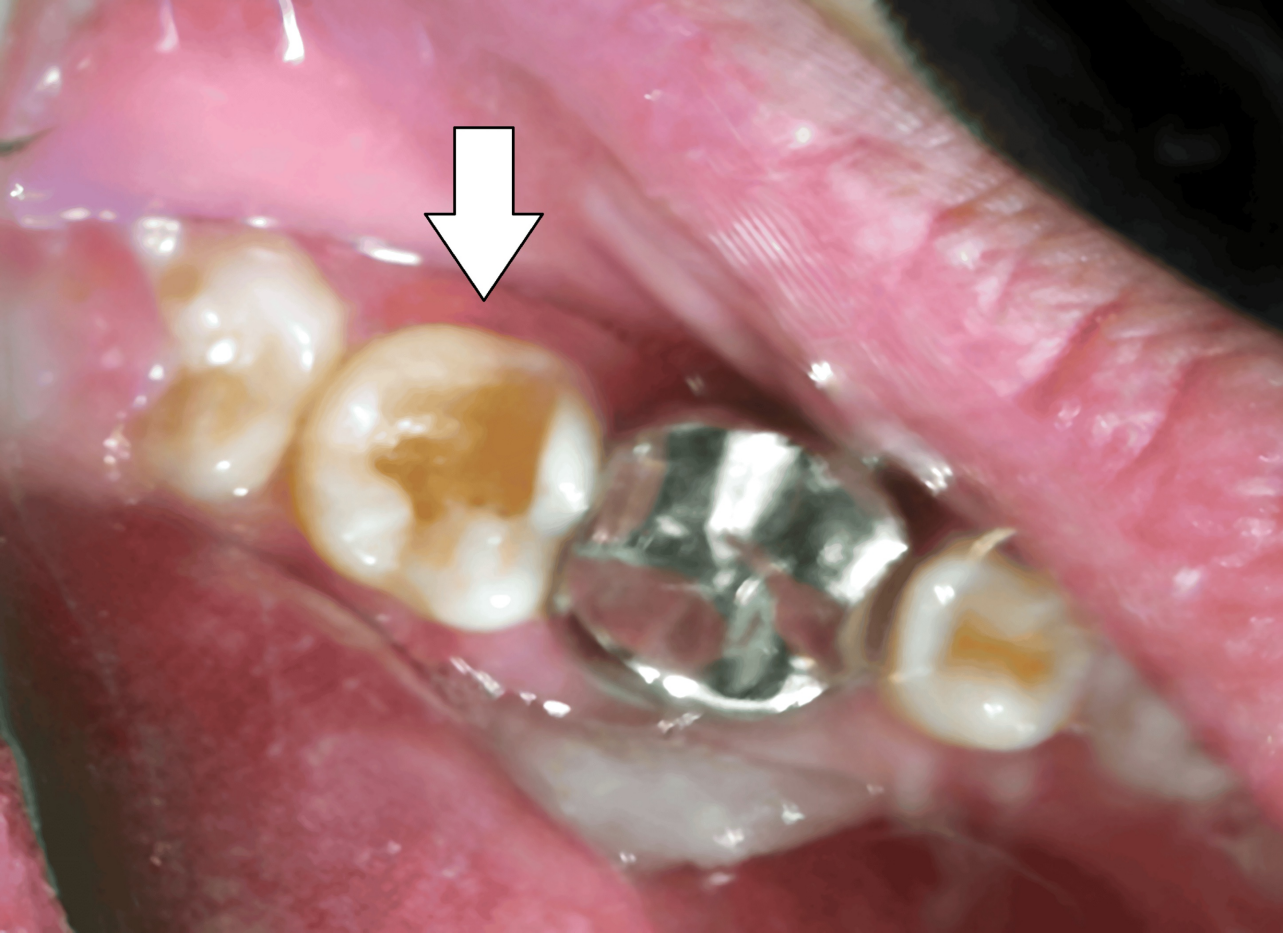
Excavation of caries along with creating buccal extension and gingival seat with 37

A silver amalgam restoration, secured with pins, was recommended, and informed consent was obtained from the patient. The procedure began with the excavation of caries using a high-speed handpiece with a BR45 round bur to remove the carious enamel and dentin, gaining access to the cavity. A slow-speed handpiece (300 rpm) with a larger round bur (No. 6 or No. 8) and a spoon excavator was used to remove infected dentin. After excavation, the buccal extension and gingival seat were prepared (Figure 1). 

**Figure 2:**
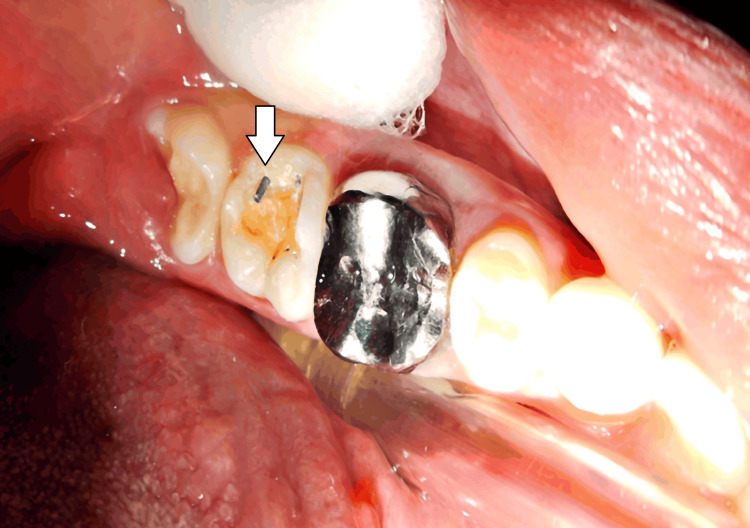
Placement of a self-threaded pin into the dentin

The cavity walls and margins were refined with a high-speed handpiece to ensure smooth and well-defined edges for better restoration retention. After the complete removal of caries, varnish and a base of zinc phosphate was applied, and slots were prepared. For retention, two pins were planned: one in the distal cusp, extending 0.5 mm into the dentin, and the other in the central groove (the floor of the cavity), extending 0.5 mm into the dentin-enamel junction. The length of pin was 6.7 mm, and for retention, a 4.7 mm pin was placed into the dentin (Figure 2).

**Figure 3:**
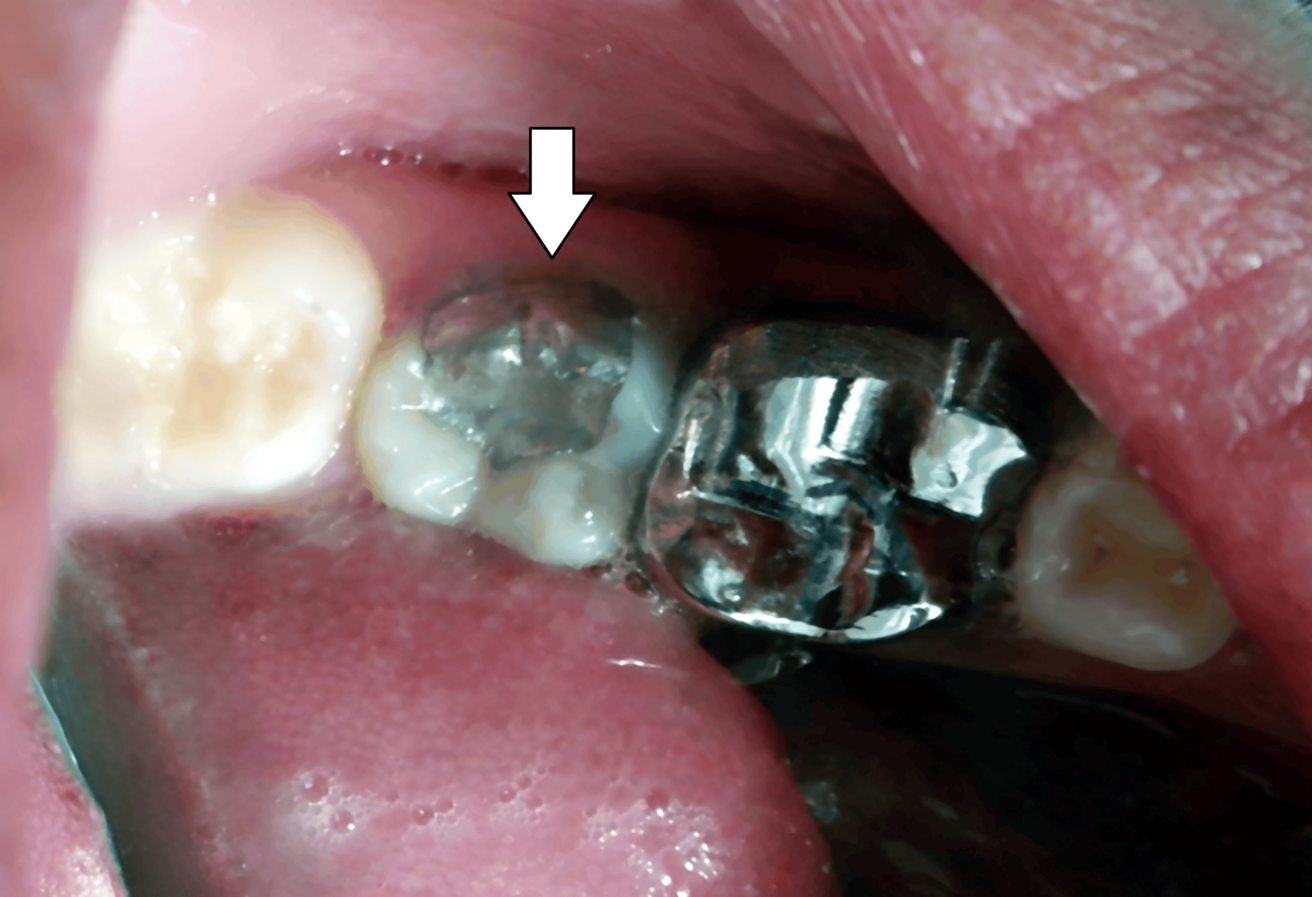
Amalgam restoration with 37

This was done using a contra-angled handpiece which offered rotational speed of 500 revolutions in a minute with the objective of placing the pin into the pin slot. As follows, the prepared tooth was fitted with a Tofflemire retainer, wedges, and a matrix band. Following the above procedures of condensing the amalgam around the pins and gradually building it up: pre-carve burnishing, carving, occlusion check, and post-carve burnishing followed the above procedures. The next day continued with finishing and polishing (Figure 3).

**Figure 4:**
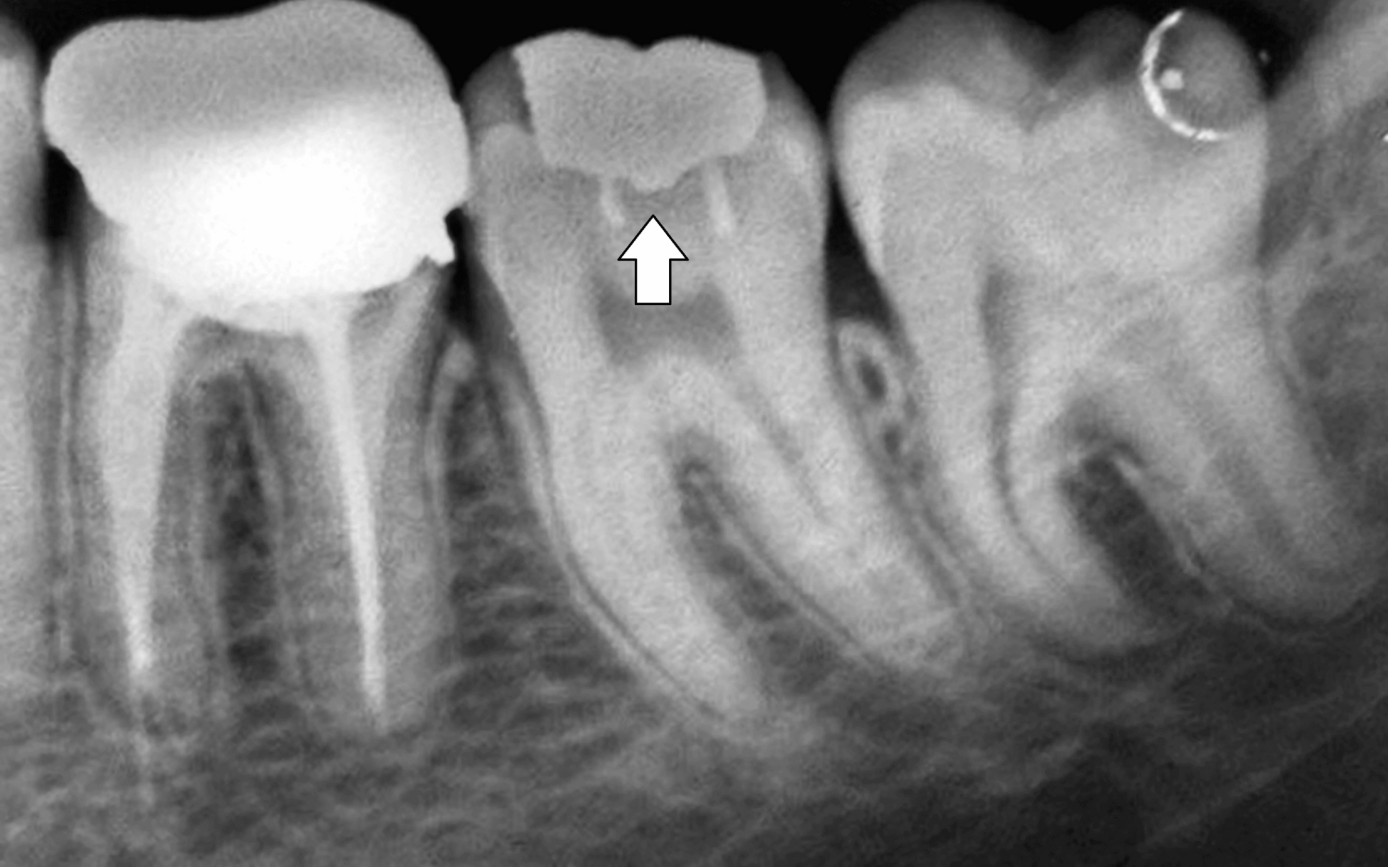
Radiographic presentation of pin-retained amalgam with respect to 37

 A radiograph was taken to check the margins of restoration (Figure 4).

## Discussion

The goal of dental restoration is to replace or restore the lost tooth structure. Enhancing dental health and the ability to chew is the primary objective of dental restoration [8]. One of the oldest restorative materials is silver amalgam which has been used in the treatment of decayed teeth, or to rebuild the tooth itself when and if needed. There are two ways through which a tooth can be restored; they are direct and indirect restoration. Direct restorations do not need the dental laboratory because they can be fully created and finished inside the oral cavity. Usually, they can be finished in a single dental appointment and do need the use of a provisional or temporary restoration. Restorations that are classified as indirect must first be made outside of the mouth and then fitted onto the prepared cavity because they cannot be fabricated into the oral cavity. Dental implants, crowns, bridges, inlays, onlays, and veneers are a few examples [9]. The case we have discussed in this report was one of the types of complex amalgam restorations. The need for complex restorations arises when there is very little bulk of remaining tooth structure available and there is no other way to help retain the restorative material in or on the tooth surface without using an alternative. Smales et al. found a 66.7% survival rate after 10 years for large, cusp-covered amalgam restorations [10].

Complex restorations require secondary retention for the survival of the restoration; for which preparation of a slot, retentive groove, box preparation, pins, and other features are prepared. Pin-retained amalgam restoration requires the placement of a pin into the dentin for secondary retention, and the restoration is done using the support of the pin. The purpose of placing pins is to secure the restorative material in a prepared tooth cavity. It is less expensive than an extra or intracoronal restoration [11].

The pins used in this case were self-threading pins, and they provide better retention of amalgam as compared to the other pins. Amalgam and composites can be used to restore the majority of teeth. However, the remaining tooth structure is limited if a significant piece of the crown is lost owing to caries or other factors. Obtaining resistance and retention form is challenging in those cases [12]. McDaniel et al. surveyed 1200 cases and highlighted that among the cuspal-coverage amalgam restorations, tooth fracture was the leading cause of the failure. They believed that one of the contributing factors for this failure was the conservative tooth preparation; they suggested to replace these fragile cusps with large amalgam restorations [13].

Ever since Markley presented his first report on the pin retention of amalgam in 1958, a lot of work has been carried out on this area. Another author who conduced a study on the retentive factors of three types of pin shank designs in dentin and amalgam was Moffa et al. in 1969. They wrote that 2 mm was the maximum length of retentive pin in the dentin and the pin in amalgam, and lastly, they said that the self-threading pin was more retentive than the others [14,15].

## Conclusions

Pin-retained amalgam restorations provide one of the strongest and most stable restoration techniques for the teeth which need more retention and stability due to a significant amount of tooth structure loss. It should be noted that these restorations are not very esthetic; however, they possess excellent occlusal strength, which means that they are long-wearing. Nonetheless, the technique should be precise in order to prevent various problems like microleakage, secondary caries, or pulp damage. Common in extensive preparations, root-filled teeth, and multi-surfaced restorations, pin-retained amalgam restorations remain an important procedure in restorative dentistry if done under strict sterile isolations.
